# Identifying shape transformations from photographs of real objects

**DOI:** 10.1371/journal.pone.0202115

**Published:** 2018-08-16

**Authors:** Filipp Schmidt, Roland W. Fleming

**Affiliations:** Department of Experimental Psychology, Justus-Liebig-University, Giessen, Hesse, Germany; Uppsala Universitet, SWEDEN

## Abstract

An important task of human visual cognition is to make inferences about properties of objects. One such property is an object's causal history: what happened to the object in its past (e.g., “this paper has been folded”). There is relatively little research on whether and how we make such inferences. We took photographs of objects from six different materials (‘wax’, ‘aluminum foil’, ‘gold foil’, ‘chicken wire’, ‘putty’, ‘cardboard’) transformed by one of four shape-altering transformations (‘folded’, ‘bent’, ‘crumpled’, ‘twisted’). By varying execution of transformation and viewpoint, we obtained 30 images of each material/transformation combination (720 images). We asked different groups of participants to: (1) name transformations and materials, (2) rate images with respect to the extent they belonged to each transformation or material class, and (3) classify images into the four transformation classes. Our results show that participants can infer transformations from object shape–with accuracy being modulated by object material. This inference of causal history from observed object shape shows that we can distinguish between intrinsic (material) and extrinsic (transformation) properties of the object. The separation of observed shape features by their causal origin (‘shape scission’) presumably involves both perceptual and cognitive abilities.

## 1. Introduction

An important capability of visual cognition is to make inferences about the properties of perceived objects. This is challenging because the proximal stimulus (e.g., retinal image) is the result of complex interactions between *intrinsic* and *extrinsic* properties. Intrinsic properties are those that tend to define what the object is; they ‘belong to’ the object and tend to persist over time; and they tend to originate from the object itself rather than external events. Examples include the object’s material composition, its typical shape, and for animate agents, self-induced motion. In contrast, extrinsic factors or events are in some sense circumstantial, variable, and come from outside the object. Examples include position, orientation, lighting conditions, viewpoint, motion caused by outside events or, as we focus on here, shape-transforming insults that deform an object (e.g., crushing, denting). Of course, not all properties can be cleanly divided into these two classes. For example, ‘usefulness’ can be thought of as relational property that depends on both internal properties of objects and external circumstances. Nevertheless, as the intrinsic properties are those that usually define what an object is and how it typically behaves, whereas extrinsic properties do not, it is widely agreed that one of the main goals of perception is to distinguish between intrinsic and extrinsic properties. Here, we focus on whether the human visual system can distinguish between material (an intrinsic property) and ‘causal history’ (specifically, the application of shape-altering transformations to an object, an extrinsic property).

**Fig 1:**
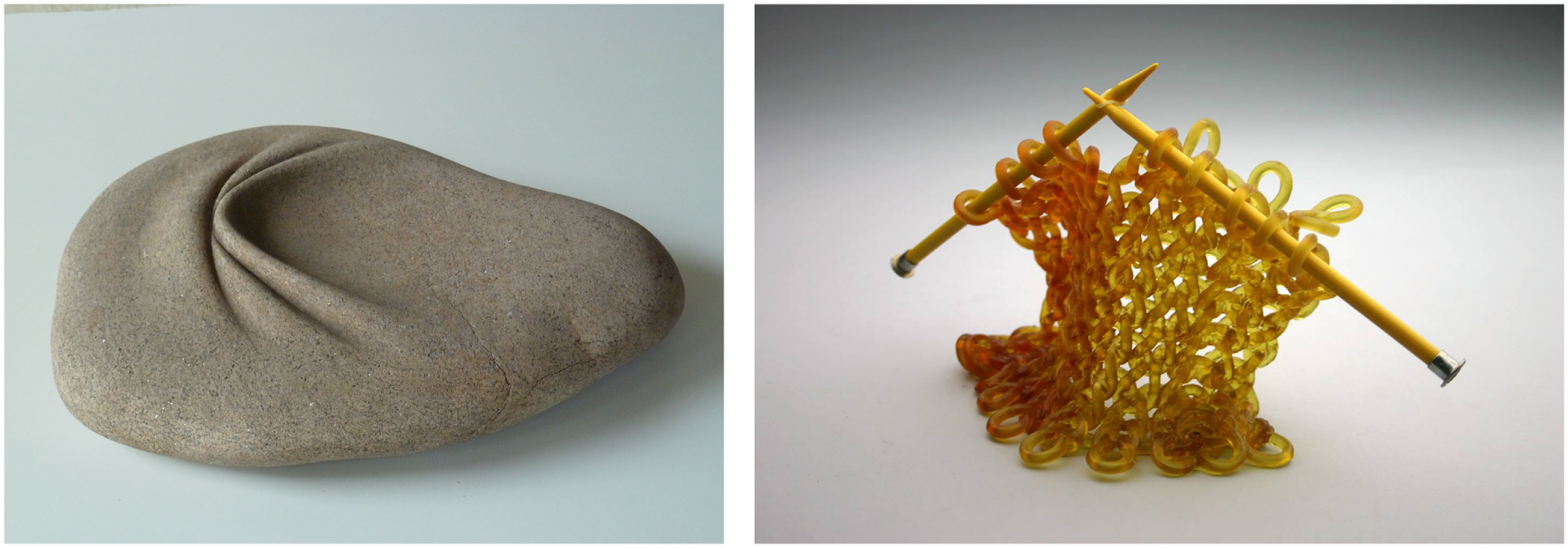
Examples of objects in which internal (material) and external (causal history) properties do not match. Object materials are granite and glass, respectively. Images are reprinted with permission (from left to right): ‘Sin título’ by José Manuel Castro López, 2010; ‘Bent’ by Carol Milne, 2014.

Potentially, many cues could be used to make inferences about intrinsic or extrinsic properties of objects; for example, the surface texture could tell us whether the object is made from wood or leather (allowing inferences about the internal property of material), or the color of a bread could tell us about whether it has been toasted (allowing inferences about the external property of causal history; e.g., [1, 2]). One of the most important cues for making inferences about object properties is shape (e.g., [3–5]). Artists create powerful demonstrations of the importance of shape by creating mismatches in perceived internal and external properties [6]. In Fig 1, we show a selection of sculptures in which objects made from rigid material (granite and glass) feature shape cues indicating causal histories that are typically only found in non-rigid materials (‘folded’ and ‘knitted together’).

However, there is relatively little experimental research about if and how we use object shape to make inferences about the causal history of objects [2, 7–15]. This is surprising given that every object gets its shape from some transformation applied to matter, and that identifying and understanding these transformations is potentially important for a wide range of perceptual and cognitive tasks, for example, for identifying objects across diverse viewing conditions or organisms across growth (object constancy) [16], predicting future behavior and motor affordances of objects, or predicting plausible variants of objects. Specifically, identifying and understanding transformations has important implications for guiding our behavior as it may facilitate inferences about object properties, such as compliance or fragility, as well as in anticipating likely future behaviors (e.g., predicting how a deformable object will move, bounce and change shape) and motor affordances (e.g., working out how to grasp and handle objects). For example, by inferring that an object has been twisted, we can make an educated guess about its internal properties (e.g., it is rather malleable and does not fracture easily) and potentially even material category (e.g., it is more likely to be a sponge brick than a real brick), which can guide us in handling the object, such as the selection of grasp locations and grip and load forces. Indeed, an object’s function, or suitability for performing some task, may change critically depending on the transformations that have previously been applied to it: a bloated can indicates the food inside has gone off; a cracked glass is unsuitable for drinking; a crumpled sheet of paper is better than a flat one for playing catch, but worse for writing a letter. Indeed, were transformations not important concepts, we most likely would not have words for them.

There is some evidence to suggest that we can make inferences about causal history–most likely by parsing and interpreting the causally significant features of shapes [11, 14, 17]. For example, Pittenger and Todd [12] showed participants a sequence of three drawings (humanoid bodies transformed by different geometric transforms) and asked them to describe what process produced this sequence. They found that only transformations inducing changes in head-to-body ratio (‘tapered stretch’) were described by most of the participants as growth. Pinna [18] demonstrates that causal history can also be inferred from single examples by providing many variations on the line drawing of a simple square, all of which lead to inferences of a different causal history (‘happenings’ in the square's past, see Fig 19 in [18]). Do these inferences depend on the transformed object being simple (like a square) or familiar (like a line drawing)?

In a series of experiments with single presentations of unfamiliar/novel objects, we have shown that participants tell apart bitten from whole objects by relying on specific shape cues [14] and that the interpretation of concavities as bites affects judgments about symmetry axis and about the front and back of objects [8]. Chen and Scholl [9] present evidence suggesting that such effects of causal history might happen at the level of visual processing (rather than at the level of cognition) by showing that observers perceived illusory motion only when a change in the contour of an object was in line with a plausible causal history (i.e., a physical intrusion)–but not when it was a simple superposition of the ‘intruding’ shape. Finally, Spröte and Fleming [7] showed that participants can use their inferences about causal history to determine and discount transformations that were applied to novel objects–participants were able to match the degree of bending between different objects and to identify objects across different degrees of bending. Again, this suggests a ‘scission’ of the representation of object shape into internal and external contributions, somewhat akin to the separation of retinal luminance values into distinct causal layers in the perception of transparency and reflectance [19–22].

However, even though there are some studies broaching the topic of causal history of objects, there is no direct evidence for visual identification of transformations from real-world objects across different materials. Here, we investigate how well we can identify the causal history of real objects and compare it to how well we can identify object material. To this end, we took photographs from samples of six different materials (*wax*, *aluminum foil*, *gold foil*, *wire*, *putty*, *cardboard*), transformed by four different physical transformations (*twisted*, *crumpled*, *bent*, *folded*). Then, we asked participants to (i) freely name the materials and transformations (Experiment 1), (ii) rate the extent to which they perceived each stimulus to be from one of the materials or produced by one of the transformations (Experiment 2), and (iii) to identify the transformations in a multiple-choice task (Experiment 3). Note that we chose transformations affecting object shape without at the same time affecting material properties (i.e., ‘folding’ vs. ‘burning’ or ‘rusting’).

We hypothesize that participants should be able to name materials and transformations, and identify materials and transformations in the subsequent experiments, above chance performance. If participants can do these tasks, we can conclude that they can visually identify certain kinds of transformation. The crossing of all levels of material and transformation lets us investigate the effect of material on the identification of transformations, and vice versa. This allows us to test if there are interactions between inferences about internal and external properties of objects. Even though our sample of stimuli is limited to particular sets of materials and transformations, we can still derive some general characteristics about the perception of causal history.

## 2. Experiment 1: Free naming task

### 2.1 Materials and methods

#### 2.1.1 Participants

15 students from the Justus-Liebig-University Giessen (7 female, *M* = 29.6 years), Germany, with normal or corrected vision participated in the experiment for financial compensation. All participants provided written informed consent, were debriefed after the experiment, and were treated according to the ethical guidelines of the American Psychological Association. All testing procedures were approved by the ethics board at Justus-Liebig-University Giessen and were carried out in accordance with the Code of Ethics of the World Medical Association (Declaration of Helsinki).

#### 2.1.2 Stimuli

**Fig 2:**
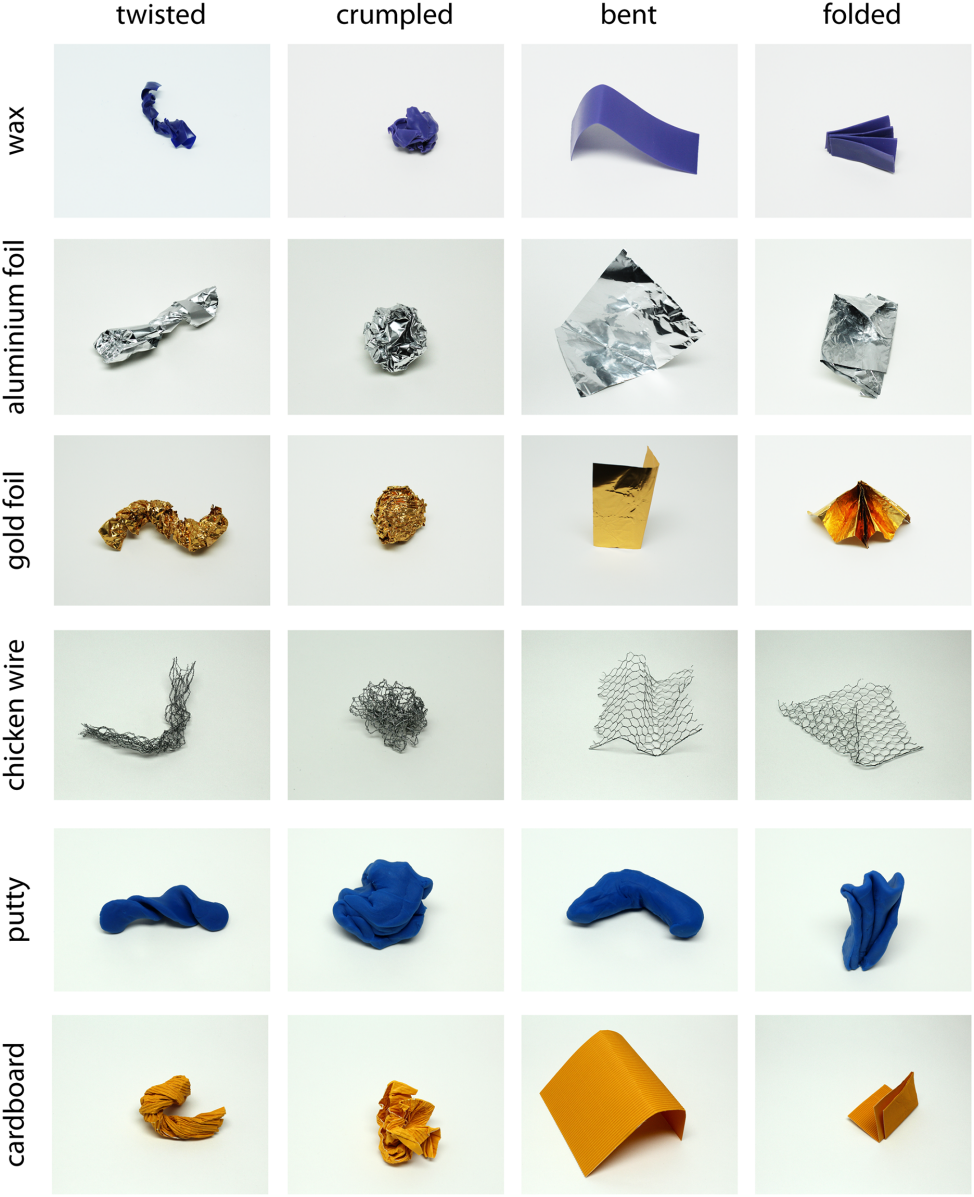
Example stimuli for all combinations of the six materials (rows) and four transformations (columns).

Stimuli were photographs of real samples of six different materials (*wax*, *aluminum foil*, *gold foil*, *wire*, *putty*, *cardboard*), transformed by four different transformations (*twisted*, *crumpled*, *bent*, *folded*). These were selected out of a larger set of materials and transformations to enable a factorial design, such that all tested transformation could be applied to all tested materials. We varied the execution of the transformation (e.g., different types of folding) and viewpoint, yielding 30 stimuli per combination of material and transformation (6 × 4 × 30 = 720 stimuli in total; see Fig 2 for example stimuli). Note that we did not use a standardized procedure for transformation execution or viewpoint variation as both were merely ways to increase stimulus number and variability and were not intended to be analyzed as an experimental variable. Objects were placed on a homogeneous white background and illuminated under standardized light conditions with a normlight, and then photographed using a Canon D600 camera with the standard kit lens in AV-mode with an ISO of 400 and an aperture of F 6.3. The resulting images were color-balanced, cropped and re-sized so that the object filled approximately the same image area across all images. All stimuli are available at https://doi.org/10.5281/zenodo.1244096.

#### 2.1.3 Procedure

For Experiment 1, we chose for each participant 24 different objects defined by the combination of six materials and four transformations with random execution/viewpoint, so that 360 stimuli out of the 720 available stimuli were part of this experiment. Stimuli were presented on a white background on a Dell U2412M monitor at a resolution of 1920 × 1200 pixels. Height and width of each stimulus on screen was approximately 30 × 22 cm (about 34.38 × 25.21° of visual angle at a monitor distance of about 50 cm). In each experimental trial, participants were presented with a single stimulus in the center of the screen and were asked to name (1) the object material, as well as (2) the process that formed the current shape of the object (i.e., causal history): “*In the following*, *you will be presented with objects of different materials*, *that were brought into their current shape by a specific process or procedure*. *Your task is to describe or name (1) the material*, *and (2) the process or procedure*. *If you can think of multiple responses*, *provide all of them*.” The presentation order of stimuli was randomized. Overall, each participant responded to four stimuli for each of the materials (across transformations), and to six stimuli for each of the transformations (across materials).

#### 2.1.4 Analysis

**Fig 3:**
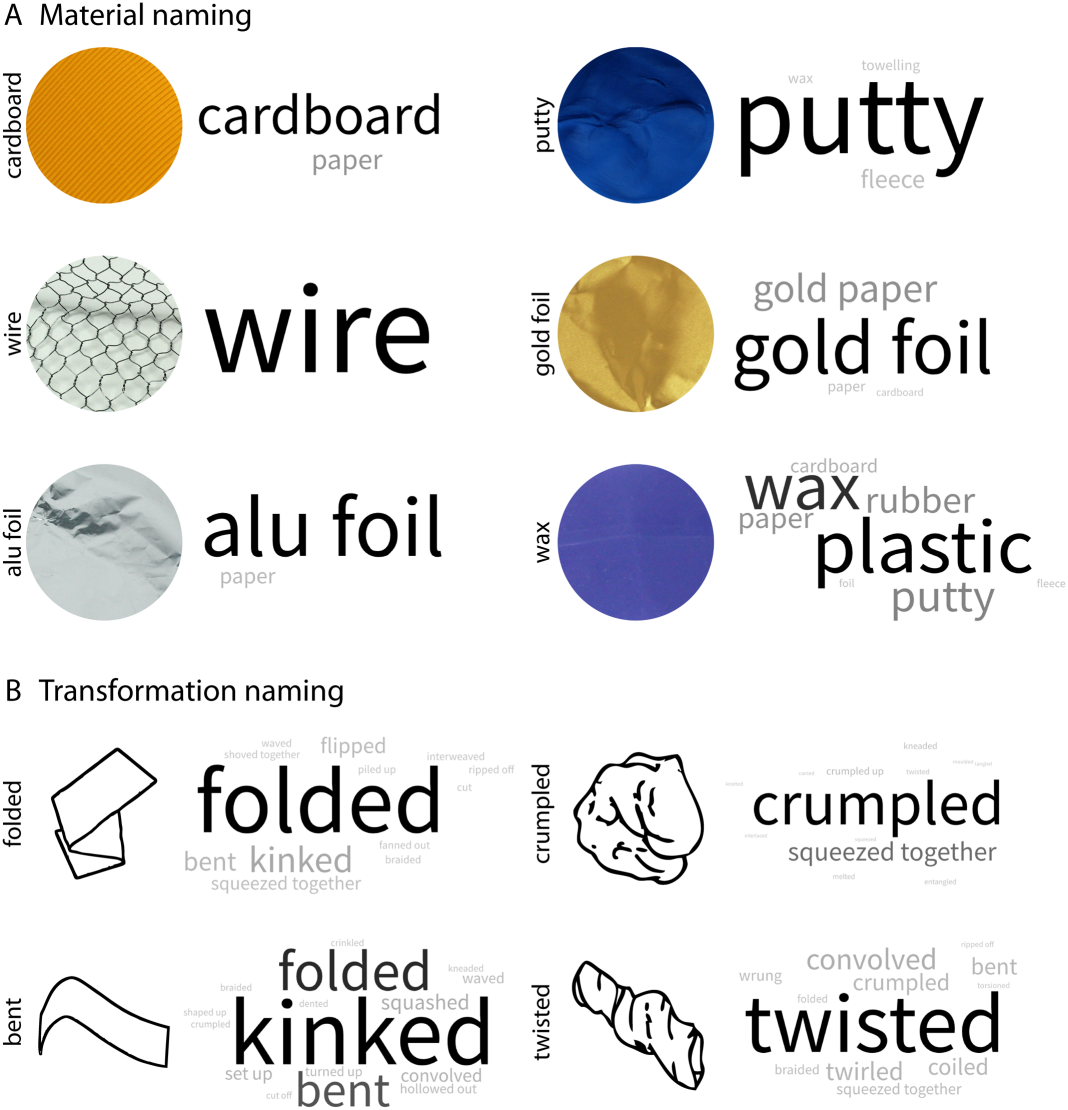
Word clouds for raw free naming task data for (A) materials, and (B) transformations. Size and saturation of naming responses is based on their relative frequency. For bar plots of raw frequencies see S1 Fig.

**Fig 4:**
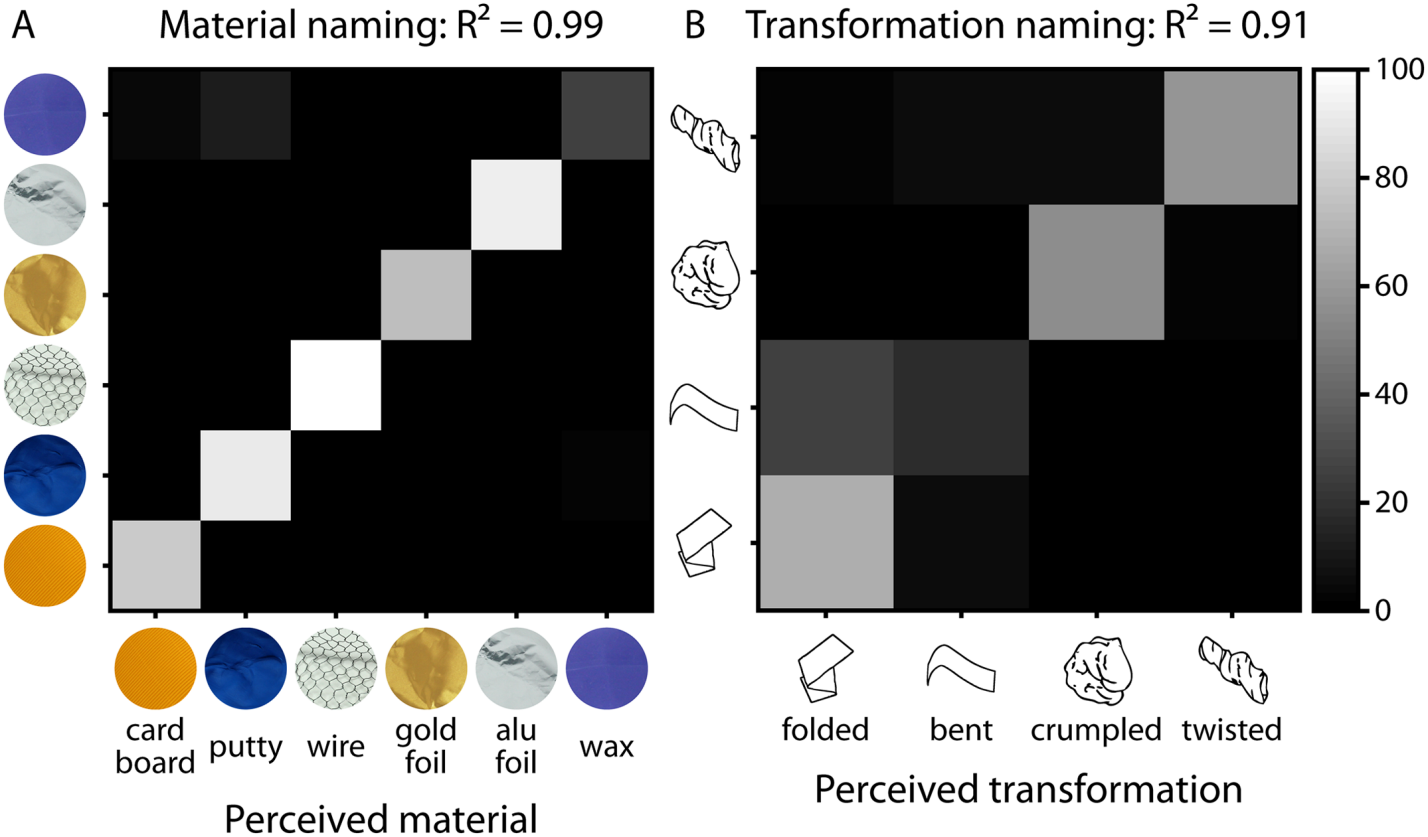
Confusion matrices for the results of the free naming tasks [percentages] for (A) material naming and (B) transformation naming. Actual materials/transformations on the y axis are plotted against perceived materials/transformations on the x axis. Note that we plot percentages of all responses, including those that are not included in the confusion matrices (e.g., material: plastic; transformation: squeezed together), so that values in the rows/columns do not add up to 100%. R^2^ values indicate how much matrices resemble a perfect prediction matrix (see text for details).

For presentation in this paper, we translated the naming data from German to English. Then, we counted frequency of names for materials and causal history (Fig 3 and S1 Fig for raw data). As we are interested in the relative frequency with which participants correctly identified materials and causal history and to what extent they mixed up the different materials/histories, we determined confusion matrices based on relative percentages (Fig 4). All data are available at https://doi.org/10.5281/zenodo.1244096.

### 2.2 Results and discussion

In Fig 3, we show word clouds for the raw naming data. For all materials but wax, the majority of naming responses was that of the actual material (Fig 3A). Equivalently, for all transformations but bent, the majority of naming responses was that of the actual transformation (Fig 3B).

We also plot confusion matrices to visualize the specific pattern of errors (Fig 4), with actual materials/transformations plotted against perceived materials/transformations. As we plot percentages of all responses, including those that are not included in the confusion matrices (e.g., material: plastic; transformation: squeezed together), values in the rows/columns do not add up to 100%. For performance evaluation, we calculate correlations between the matrices and a perfect prediction matrix (all zeros but a diagonal of ones). Additionally, we report as a performance measure the inverse distance between participant's response matrices and the prediction matrix *perf = 1-mean(abs(responseMatrix-predictionMatrix)* with *perf* = [0, 1] where lower values indicate lower performance and higher values indicate higher performance with chance level *perf* = 0.5. For material naming, performance of participants was very close to optimal (R^2^ = 0.99; *perf* = 0.99, 95% CI [0.98, 1.00]; compared to 100,000 bootstrapped random prediction matrices: R^2^ = 0.00; *perf* = 0.51, 95% CI [0.41, 0.61]), and for transformation naming, performance was also clearly above chance (R^2^ = 0.91; *perf* = 0.90, 95% CI [0.84, 0.97]; compared to 100,000 bootstrapped random prediction matrices: R^2^ = 0.00; *perf* = 0.57, 95% CI [0.43, 0.72]).

## 3. Experiment 2: Rating task

### 3.1 Materials and methods

#### 3.1.1 Participants

Two new group of 15 students from the Justus-Liebig-University Giessen (8 female, *M* = 26.5 years; and 11 female, *M* = 28.1 years), Germany, with normal or corrected vision participated in the experiment for financial compensation. All other details were the same as in Experiment 1.

#### 3.1.2 Stimuli

Stimuli were the same as in Experiment 1.

#### 3.1.3 Procedure

For Experiment 2, each participant was presented with 168 stimuli defined by the combination of six materials, four transformations and seven executions/viewpoints. Details of presentation were the same as in Experiment 1. Each of the 168 stimuli were presented to each participant six (first group) or four (second group) times, in six or four blocks of 168 trials. In each trial, participants were presented with a single stimulus in the center of the screen and a rating bar at the bottom of the screen.

In the first group of 15 participants, the rating bar was labeled with the name of one of the materials (‘wax’, ‘aluminum foil’, ‘gold foil’, ‘wire’, ‘putty’, ‘cardboard’), and its ends with “not true” and “true”. In the second group of 15 participants, it was labeled with the name of one of the transformations (‘twisted’, ‘crumpled’, ‘bent’, or ‘folded’) and its ends with “not true” and “true”. They received the following instructions: “*Look closely at each object and judge the extent to which you think it is made from the given material*. *Use the mouse to choose a position on the rating bar*. *Take as much time as you need for an accurate response*.” (first group); and “*Look closely at each object and judge the extent to which you think it was brought into its current shape by the given process*. *Use the mouse to choose a position on the rating bar*. *Take as much time as you need for an accurate response*.” (second group).

Within each of the blocks, participants rated all of the stimuli with respect to one of the material/transformation names (e.g., in the first block, a given participant rated all 168 stimuli according to the extent to which they appeared twisted). The order of blocks and the presentation order of stimuli within blocks was randomized.

#### 3.1.4 Analysis

For the results of the rating task, we looked at the correlations between the matrices and a perfect prediction matrix (cf. Experiment 1). Additionally, we calculated for each group a repeated-measure ANOVA (rmANOVA) for rating accuracy with factors of material (6) and transformation (4) within blocks. Ratings were averaged across variations in execution of transformations and viewpoint. We report Huynh-Feldt-corrected degrees of freedom and *p* values. All data are available at https://doi.org/10.5281/zenodo.1244096.

### 3.2 Results and discussion

**Fig 5:**
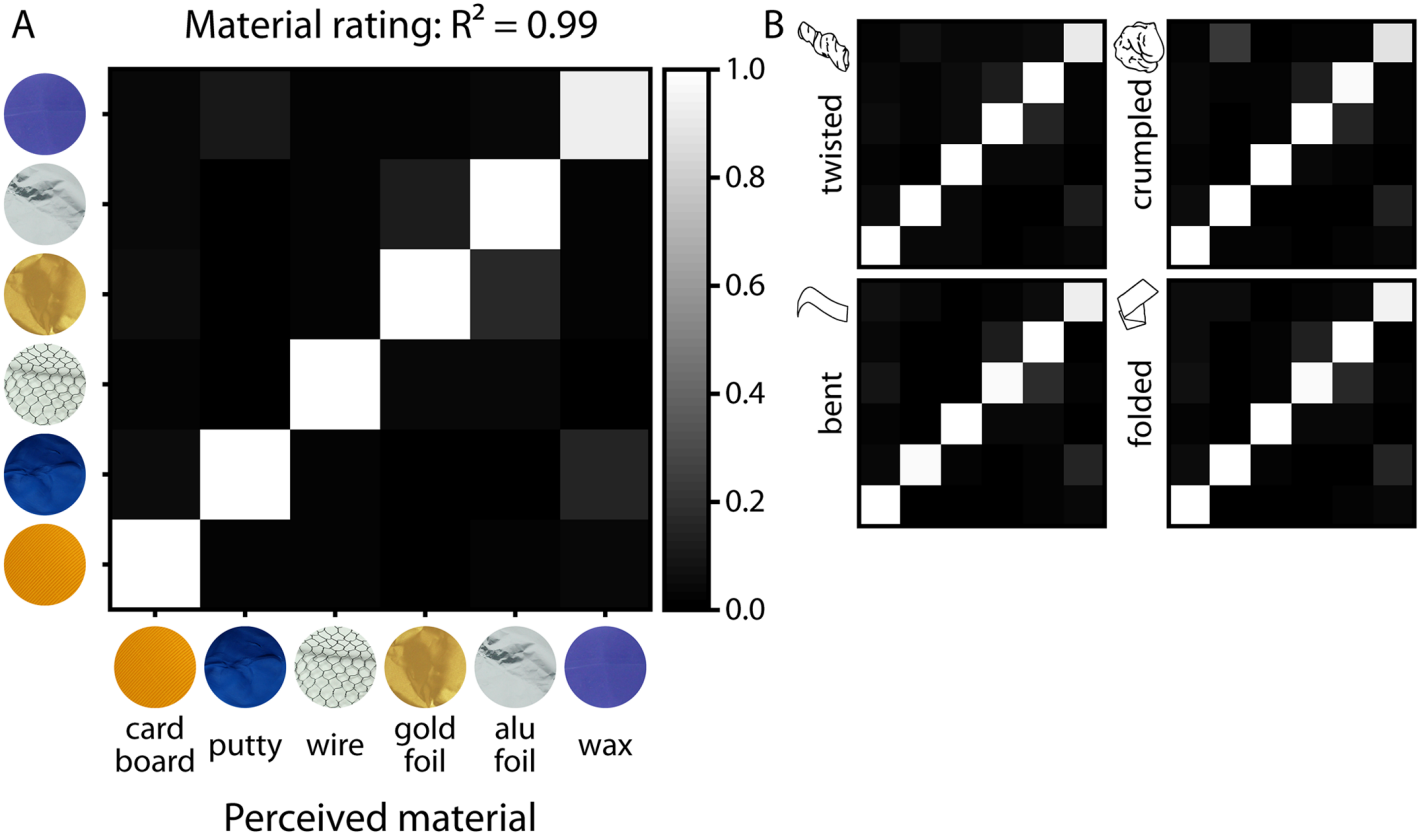
Confusion matrices for the results of the material rating task [accuracy] for (A) material ratings and (B) material ratings per transformation class. Actual materials on the y axis are plotted against perceived materials on the x axis. R^2^ values indicate how much matrices resemble a perfect prediction matrix (cf. Experiment 1).

For material ratings, performance of participants was very close to optimal (R^2^ = 0.99; *perf* = 0.96, 95% CI [0.95, 0.97], Fig 5; compared to 100,000 bootstrapped random prediction matrices: R^2^ = 0.00; *perf* = 0.54, 95% CI [0.44, 0.64]). The average correlation between all individual confusion matrices (calculated per material, material rating, and transformation) was r = 0.92, indicating high consistency between observers. The rmANOVA showed that accuracy was by trend affected by material [*F*(1.37,19.11) = 3.84, *p* = .054], with post-hoc contrasts showing that this was driven by the lower performance for wax compared to the other materials (S1 Table). There was no effect of transformation (S2 Table) or an interaction of material and transformation.

**Fig 6:**
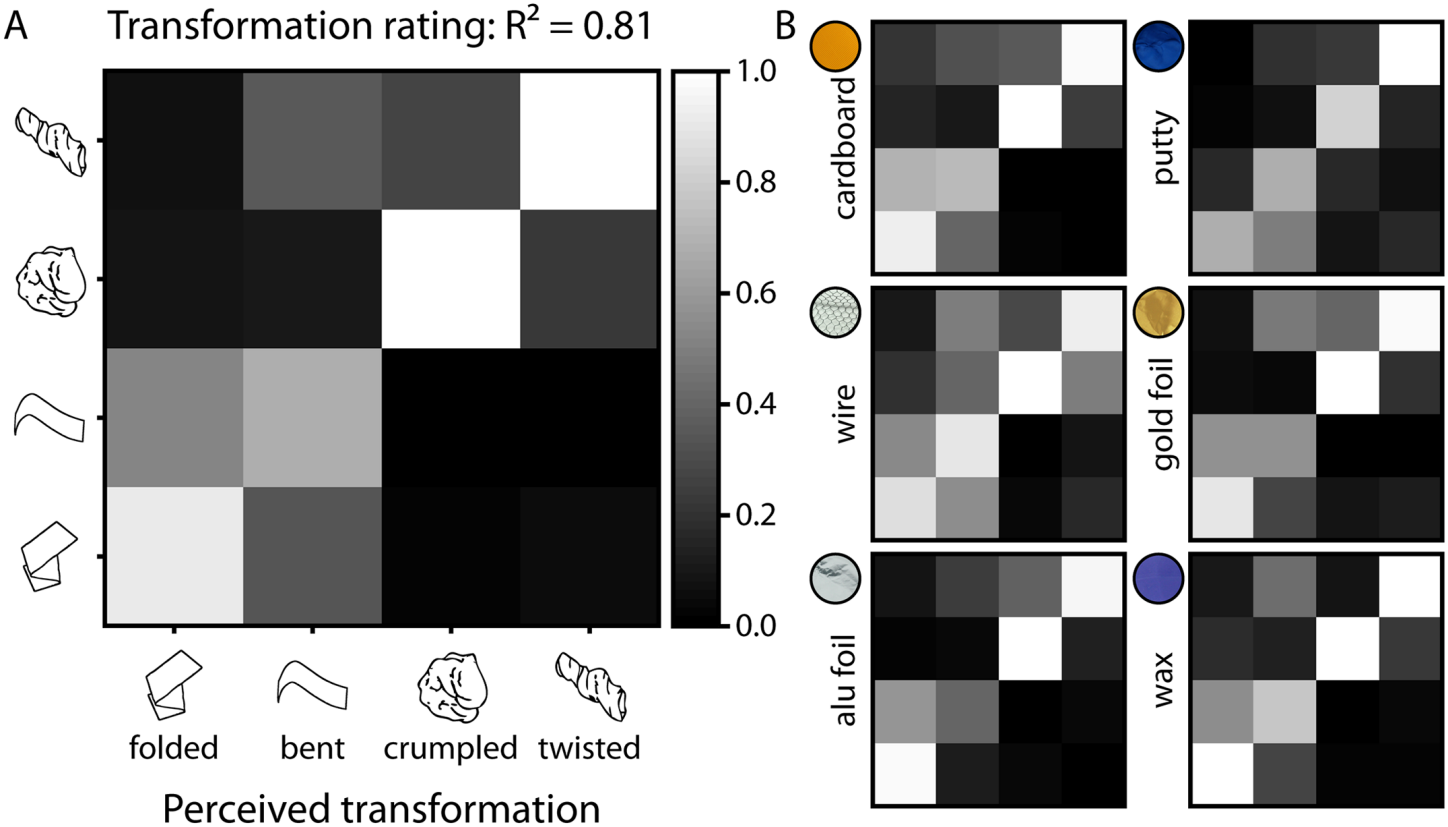
Confusion matrices for the results of the transformation rating task [accuracy] for (A) transformation ratings and (B) transformation ratings per material class. Results are averaged across observers and execution of transformation and viewpoint. Actual transformations on the y axis are plotted against perceived transformations on the x axis. R^2^ values indicate how much matrices resemble a perfect prediction matrix (cf. Experiment 1).

For transformation ratings, performance was also clearly above chance (R^2^ = 0.81; *perf* = 0.81, 95% CI [0.73, 0.88], Fig 6; compared to 100,000 bootstrapped random prediction matrices: R^2^ = 0.00; *perf* = 0.64, 95% CI [0.50, 0.77]). The average correlation between all individual confusion matrices (calculated per transformation, transformation rating, and material) was r = 0.78, indicating a substantial consistency between observers. The rmANOVA showed that accuracy was affected by the type of transformation [*F*(1.68,23.49) = 26.11, *p* < .001], mainly driven by the lower performance for bent (and somewhat by the lower performance for folded) compared to the other transformations (S3 Table). Also, there was an effect of material [*F*(2.37,33.19) = 6.10, *p* = .004], driven by the higher performance for cardboard and wax (S4 Table) compared to the other materials, as well as an interaction of transformation and material [*F*(8.60,120.35) = 8.64, *p* < .001], where for example performance for bent was especially low with gold foil and aluminum foil compared to with other materials (S5 Table).

**Fig 7:**
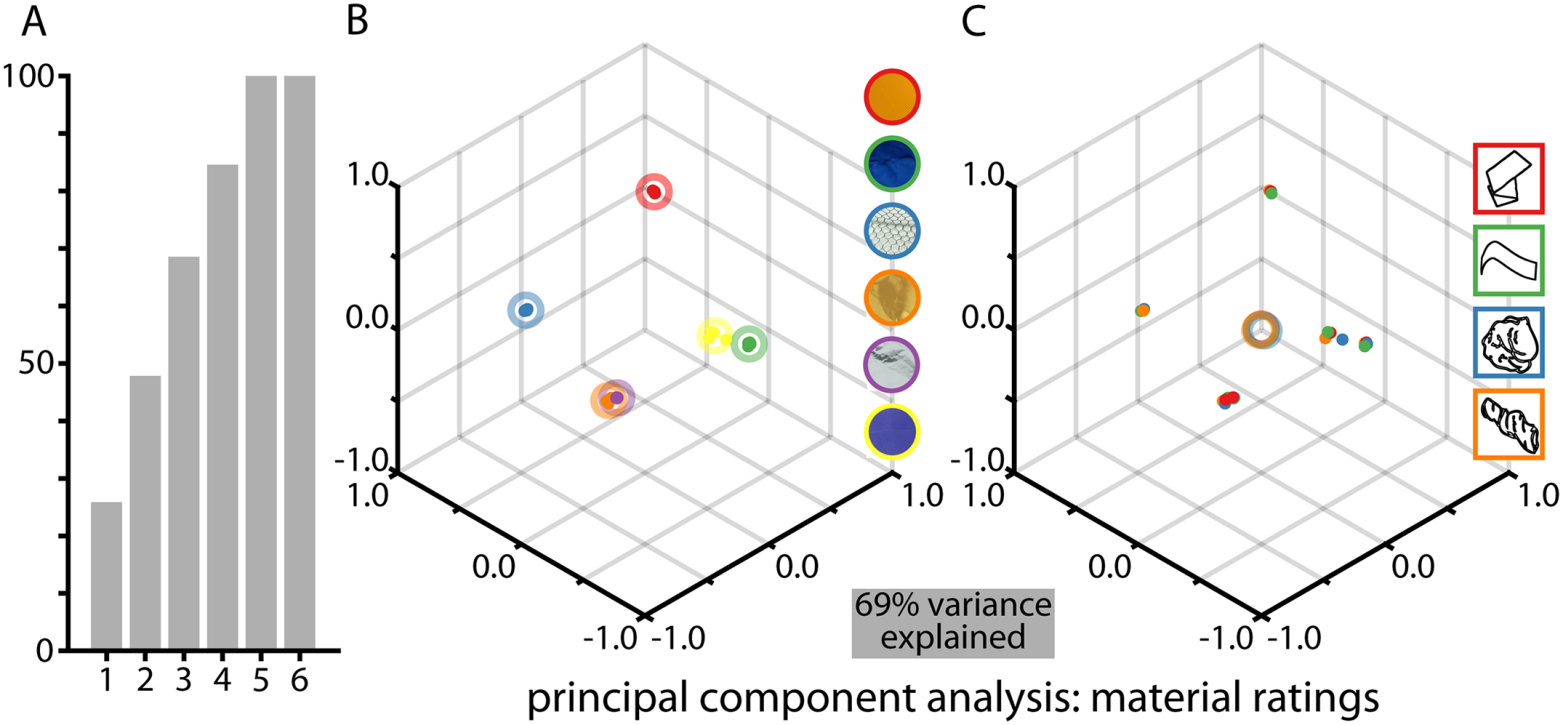
PCA for the results of the material rating task. **(A) shows the explained variance as a function of the number of PCA components**. We also plot the rating responses in PCA space, labeled by (B) material, or (C) transformation. Single dots show responses averaged per transformation class and circles show grand averages of these responses.

Another way to look at the data is in terms of the extent to which participants can tease apart the material properties and transformation, in the two complementary tasks with the same stimuli (material and transformation ratings). As we have multiple ratings for every stimulus, we can consider each stimulus as a point in the high-dimensional space defined by the set of responses. To visualize these, we performed a principal component analysis (PCA) on responses in the material rating task, averaged per transformation, transformation rating, and material (Fig 7), and plotted the rating responses in the space spanned by the first three principal components. The plot reveals that the data are systematically organized when labeled by material (Fig 7A), but not at all when labeled by transformation (Fig 7B), indicating that judgments are dominated by the material class of the stimuli.

**Fig 8:**
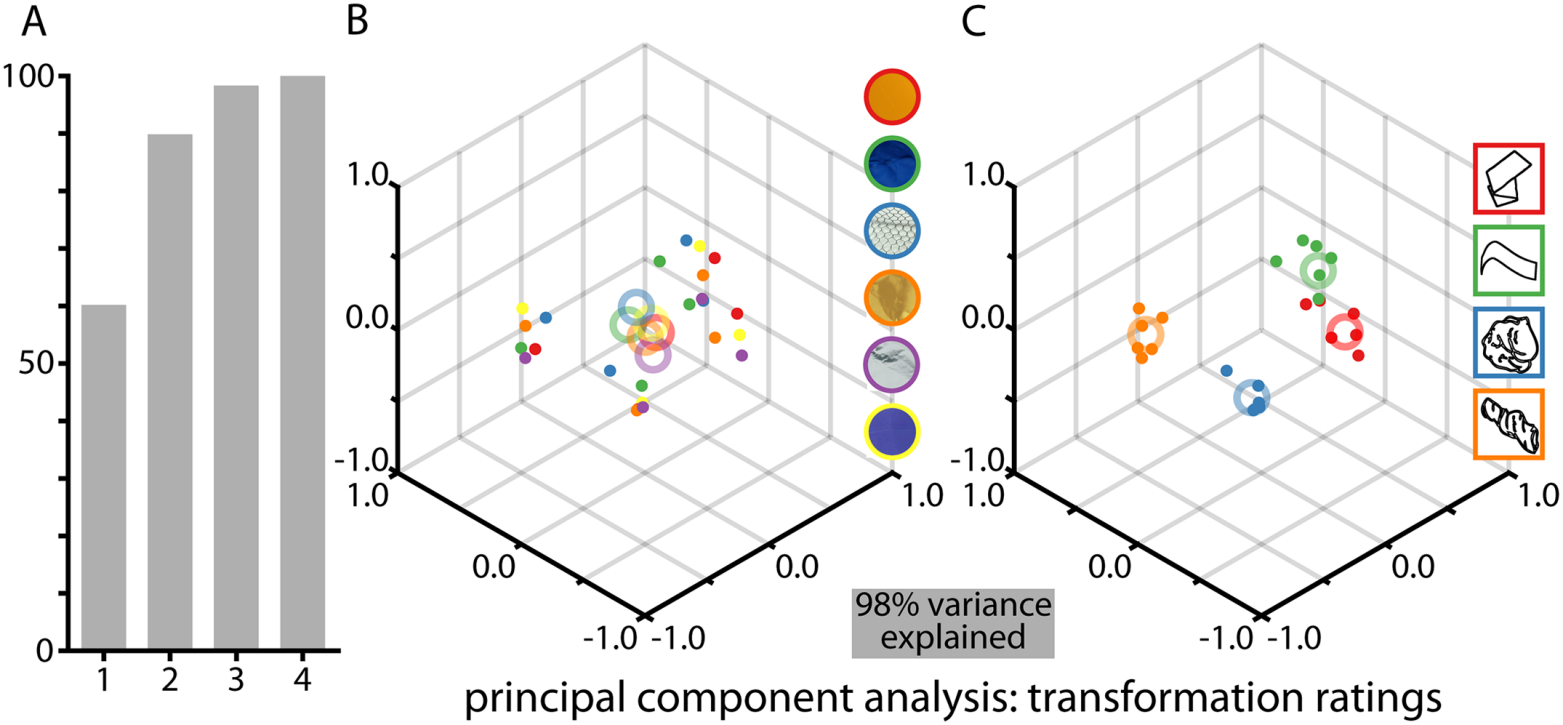
PCA for the results of the transformation rating task. **(A) shows the explained variance as a function of the number of PCA components**. We also plot the rating responses in PCA space, labeled by (B) material, or (C) transformation. Single dots show responses averaged per material class and crosses show grand averages of these responses.

This is exactly the other way around for the transformation instructions (Fig 8): when plotting the PCA solution for the first three factors, data are neatly organized when labeled by transformation (Fig 8B) but not when labeled by material (Fig 8A).

Together, Figs 7 and 8 indicate that participants are excellent at separating the internal and external properties of objects, and can selectively respond to either material or transformation with negligible cross-talk between the two.

Finally, to figure out which features participants might use to infer transformations, we subjected all of our 720 stimuli to a final experiment, in which we specifically sought to identify from the complete stimulus set those that were most often mislabeled (i.e., where participants most often inferred a different transformation from the physical one).

## 4. Experiment 3: 4-way categorization task

### 4.1 Materials and methods

#### 4.1.1 Participants

A new group of 15 students from the Justus-Liebig-University Giessen (9 female, *M* = 31.0 years), Germany, with normal or corrected vision participated in the experiment for financial compensation. All other details were the same as in Experiment 1.

#### 4.1.2 Stimuli

Stimuli were the same as in Experiment 1.

#### 4.1.3 Procedure

For Experiment 3, each participant was presented with all 720 stimuli defined by the combinations of (6) materials, (4) transformations and (30) executions/viewpoints. Details of presentation were the same as in Experiment 1. In each experimental trial participants were presented with a single stimulus in the right half of the screen and the four written transformation names in the left half of the screen (‘twisted’, ‘crumpled’, ‘bent’, and ‘folded’). For each stimulus, they choose the transformation fitting the stimulus most by ticking the box next to the name: “*Look closely at each object and judge how you think the object was brought into its current shape*. *Use the mouse to choose between four options*: *‘twisted’*, *‘crumpled’*, *‘bent’*, *and ‘folded’*. *Select the option you consider the most accurate*.”. The presentation order of stimuli was randomized.

#### 4.1.4 Analysis

For the results of the 4-way categorization task, we looked at the correlations between the matrices and a perfect prediction matrix (cf. Experiment 1) and calculated a rmANOVA for rating accuracy with factors of material (6) and transformation (4). Ratings were averaged across variations in execution of transformations and viewpoint. We report Huynh-Feldt-corrected degrees of freedom and *p* values. All data are available at https://doi.org/10.5281/zenodo.1244096.

### 4.2 Results and discussion

**Fig 9:**
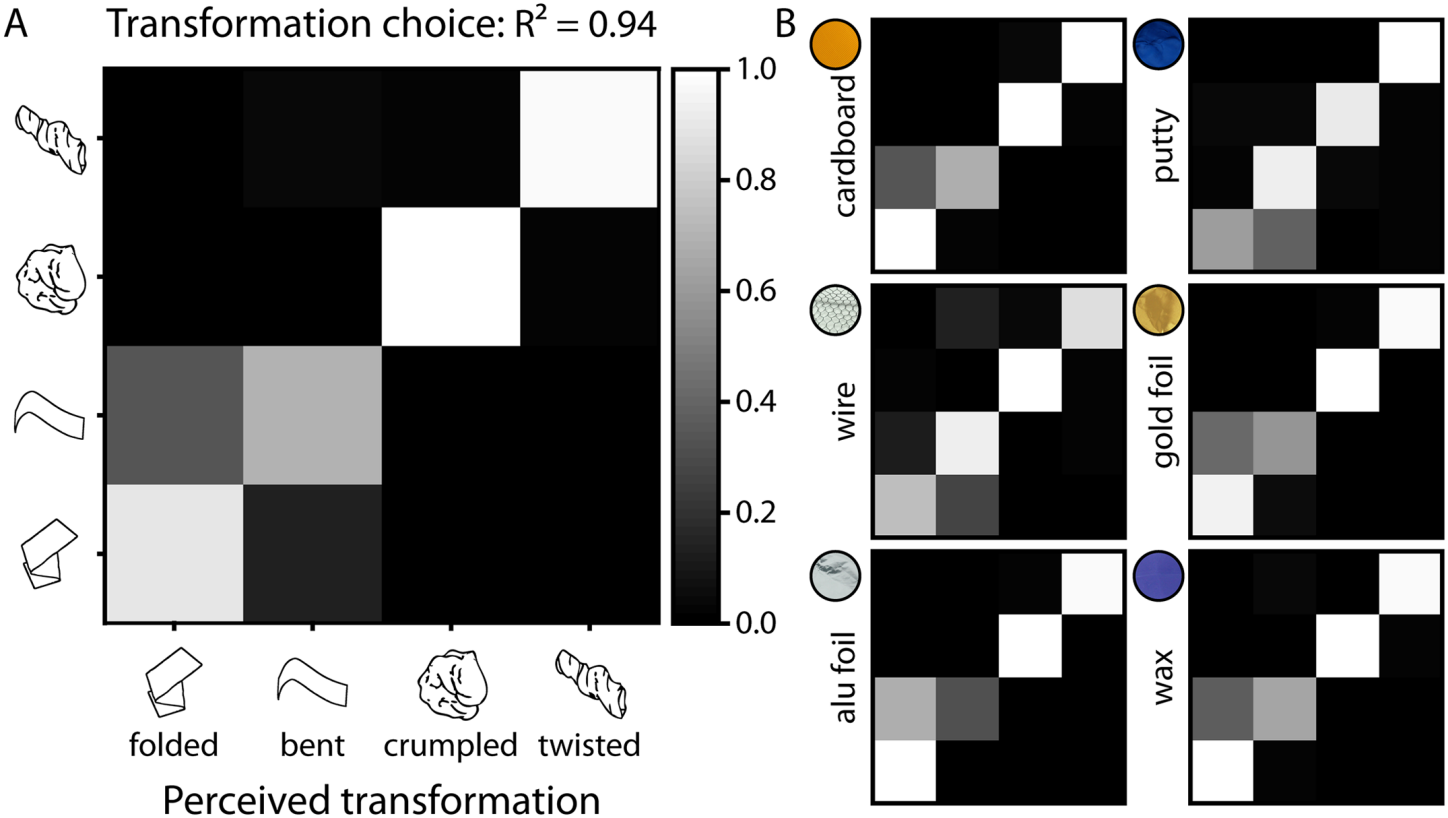
Confusion matrices for the results of the 4-way categorization task [accuracy] for (A) transformation choices and (B) transformation choices per material class. Results are averaged across observers and execution of transformation and viewpoint. Actual transformations on the y axis are plotted against perceived transformations on the x axis. R^2^ values indicate how much matrices resemble a perfect prediction matrix (cf. Experiment 1).

Again, performance of participants was clearly above chance in identifying the physical transformation (R^2^ = 0.94; *perf* = 0.93, 95% CI [0.88, 0.98], Fig 9; compared to 100,000 bootstrapped random prediction matrices: R^2^ = 0.00; *perf* = 0.55, 95% CI [0.40, 0.70]). The average correlation between all individual confusion matrices (calculated per transformation and material) was r = 0.92, indicating high consistency between observers. The rmANOVA replicated the findings from Experiment 2. Accuracy was affected by the type of transformation [*F*(1.51,21.07) = 30.82, *p* < .001], again driven by the lower performance for bent (and somewhat by the lower performance for folded) compared to the other transformations (S6 Table). The effect of material [*F*(3.44,48.13) = 3.39, *p* = .021] was driven by the lower performance for aluminum foil (S7 Table). Finally, we found an interaction of transformation and material [*F*(5.06,70.88) = 20.78, *p* < .001], where for example performance for bent was lower with aluminum foil, and for folded was lower with putty (S8 and S9 Tables).

## 5. General discussion

We set out to test whether observers can identify transformations of real-world objects from photographs (*twisted*, *crumpled*, *bent*, *folded*), across different materials (*wax*, *aluminum foil*, *gold foil*, *wire*, *putty*, *cardboard*). To this end, we asked participants to name materials and transformations, rate the extent to which they perceived each stimulus to be from one of the materials or produced by one of the transformations, and finally to identify transformations in all of our stimuli in a multiple-choice task.

### 5.1 Perceiving materials

In line with previous work [23], we find that materials were mostly named with high consistency across participants–with the exception of ‘wax’ which was often mistaken for some plastic material. As we use a range of visual features to identify materials (including shape, texture and color [2, 24]), the confusions might be driven by the fact both wax and plastic are rather heterogeneous material classes, with overlapping internal properties as well as no strongly established stereotypical color. It would be interesting to test the extent to which using typical and atypical colors could alter performance. We would hypothesize that performance for materials with a strongly established stereotypical color would be more strongly affected by changing the color than materials which are routinely encountered in a wide variety of colors.

### 5.2 Perceiving transformations

We find that even in free naming of transformations there is a remarkable consistency across participants: most participants describe twisted objects as having been twisted, crumpled objects as having been crumpled, and folded objects as having been folded. However, this is different for bent objects: even though they were also often described as having been bent, more often they were described as having been kinked or folded.

This shows that we can identify external properties of objects (i.e., their causal history) just as we can identify internal properties (i.e., their material) from photographs. In other words, when transformations leave salient visible traces in an objects’ appearance, we can identify those transformations [8, 10, 11, 18]. To put this in different terms, while a visual object category, such as ‘cup’, is the visual analogue of a *concrete noun*, a visually identified transformation—like ‘twisted’—is the visual analogue of an *adjective* or *verb*. This is supported by evidence from the child development literature. For example, 2-year-old children seeing dynamic scenes (e.g., a man waving a balloon) tend to map novel nouns (e.g., ‘larp’) to object categories (e.g., balloons) and novel verbs (e.g., ‘larping’) to event categories (e.g., waving event) [25]. Equivalently, 3-year-old children group objects based on superordinate characteristics (e.g., animals vs. food) when objects are labelled with nouns (e.g., ‘momos’), but group objects based on subordinate characteristics (e.g., relying on colour or texture; red grapes vs. green grapes) when objects are labelled with adjectives (e.g., ‘mom-ish’ ones) [26]. Note that these differences could also be used to instruct observers: when asking them to judge objects labelled with adjectives (‘red’, ‘furry’), they might tend to rely on object material (using color and texture cues); when asking them to judge objects labelled with passive verbs (‘twisted’, ‘crumpled’), they might rely on object transformations (using shape features to infer the dynamic event history).

We also found that identification of causal history was not uniform across transformations: when testing for confusions between physical and perceived transformations, we find more confusions between bending and folding compared to between the other transformations (Experiments 2 and 3). This presumably reflects inherent ambiguities in the effects of these transformations on observed shape, which we discuss below.

Probably the most important findings of the experiments are those reported in Figs 7 and 8, namely that the visual system can tease apart the complementary contributions of material and transformation to the observed image. The stimuli were identical in the two tasks—only the instructions differed, and depending on whether the participants judged material or transformation, the stimuli were grouped accordingly. This suggests that participants likely use distinct and complementary features for making these two kinds of judgment. Again, there is evidence from child development research that children can access different levels of shape representation for categorization, depending on task and context. When presented with one standard object of a particular global shape and texture, 4-year-old children group a novel object based on shape rather than texture; however, when presented with two standard objects of different shapes but same textures, they group a novel object based on texture rather than shape [27]. Equivalently, we argue that we are able to access different shape features, depending on the task at hand: when judging transformations, participants might use global shape features, while when judging materials, participants might use local, texture-like shape features.

Of course, there are limits to teasing apart contributions of material and transformation. All physical objects and materials end up with particular shapes due to some kind of generative process (such as manufacture but also biological growth or self-organization) and often the transformations involved do not leave traces in the shape of the object. For example, it is very difficult for most of us to infer the processes and forces that were applied to a computer keyboard to bring it into its current form. The same is true for many other objects. Indeed, for familiar objects, it seems likely that transformations are only perceived as such when they represent a deviation from the typical shape characteristics of the class: the rim and opener of a crumpled can are not seen as due to transformations, but the creases caused by the crumpling process are. At the same time, it is often still possible to infer intrinsic (e.g., material) properties of objects even when we are not subjectively aware of the causal history that brought them into being. There are also (more infrequent) cases in which it is the other way around. For example, we might see something twisted without being able to tell whether the material is wool or polyester. Finally, the inference of intrinsic and extrinsic properties can also inform each other. When we see a piece of cloth flapping in the wind, the type of shape transformation tells us whether the textile is a stiff waxed cloth or flexible silk.

### 5.3 Diagnostic shape features likely play a central role in the pattern of responses

**Fig 10:**
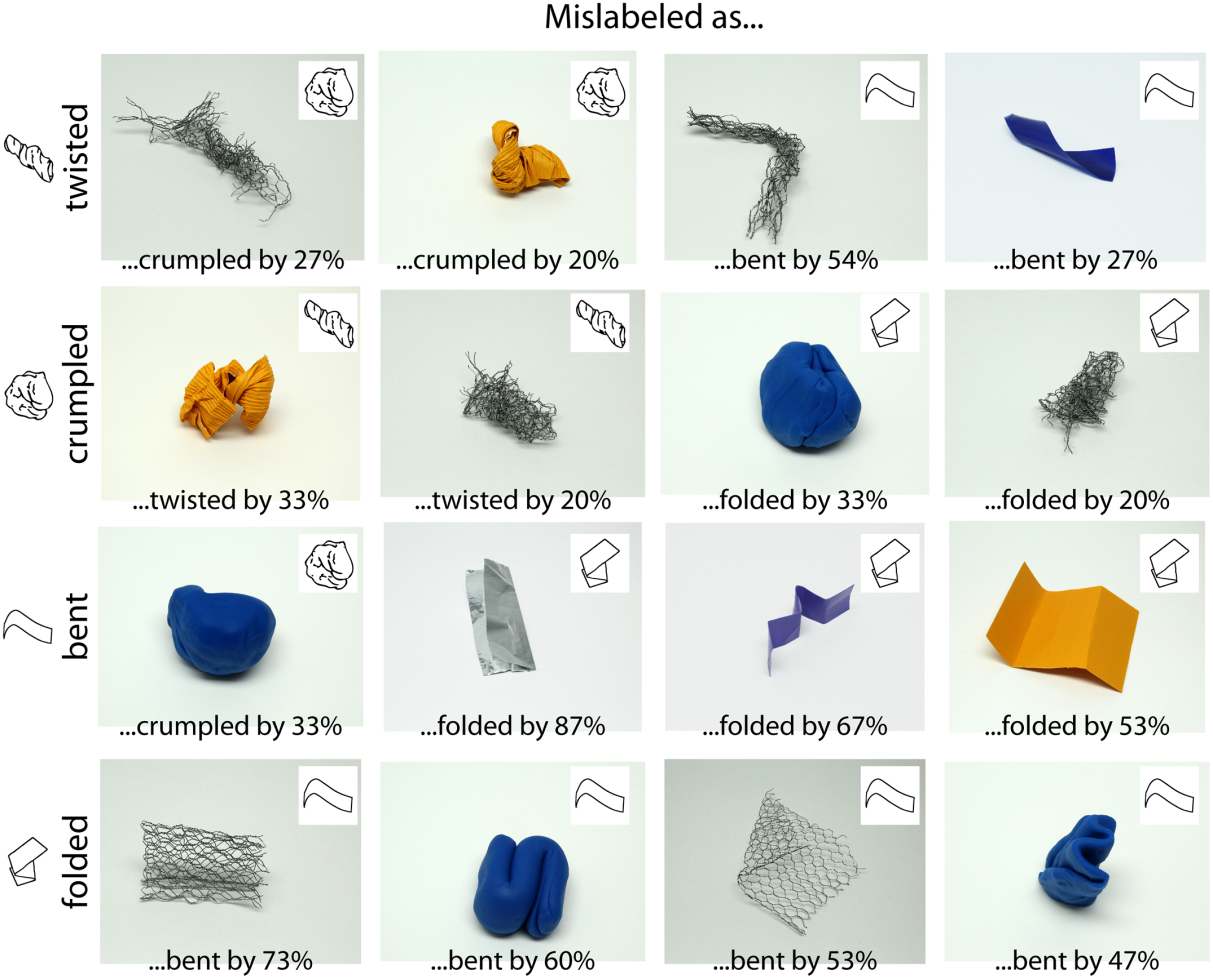
Example findings of Experiment 3. We show stimuli where a substantial percentage of participants inferred the wrong transformation (rows).

Much as we use particular visual features to identify materials (e.g., texture, color [2, 24]), it seems that observers use particular shape features when identifying transformations. Even without a formal (image-computable) analysis, this becomes obvious when inspecting those stimuli where participants most often inferred the wrong transformation (Fig 10).

Looking at the stimuli in Fig 10 and at stimuli from which the transformation was identified correctly by the majority of participants (for example those in Fig 2), we derived a tentative list of shape features that define the respective transformations (Table 1). Confusions between transformations occur when an object that was transformed by a particular physical transformation does not express the shape features which perceptually define that transformation–or rather expresses features of a different transformation. For example, bent objects are perceived as folded when the object's main axis does not follow a smooth curvy path, but rather when the object is distinguished into parts that meet at sharp angles (third row in Fig 10). Vice versa, folded objects are perceived as bent when their main axis follows a smooth curvy path, and they do not show distinct folds (fourth row in Fig 10).

**Table 1:** Transformations and corresponding local and global shape features.

	shape features	
transformations	locally	globally
twisted	*parallel spiralling creases*	*elongated shape*
crumpled	*many*, *non-parallel creases*	*spherical shape*
bent	*no sharp angles*	*main axis follows smooth curvy path*
folded	*distinct fold(s)*	*parts meeting at (acute) angles at local folds*

The list is not intended to be anyway exhaustive but should rather exemplify how a description of physical transformations might be reflected in perceptually relevant shape features.

We suggest that the perception of transformation is similar to object perception in the sense that there are *prototypical* transformations (much as there is a prototypical bird, with characteristics that are typical of birds). For example, a prototypical crumpling is defined by shape features that are obtained when crumpling is applied to a material like paper (rather than those obtained when ‘crumpling’ an atypical material, like a piece of wood). This idea of prototypical transformations probably has two components. First, some transformations are simply more frequently applied to certain materials, so the prototypical form of the transformation is associated with the features that emerge when the transformation is applied to those materials. Second, it is also the case that transformation-diagnostic features are expressed more saliently when the transformation is applied to some materials and objects than others. For example, a runny fluid, like yoghurt, will not retain the traces of being stirred for very long, while a thick paste, like cream cheese will.

**Fig 11:**
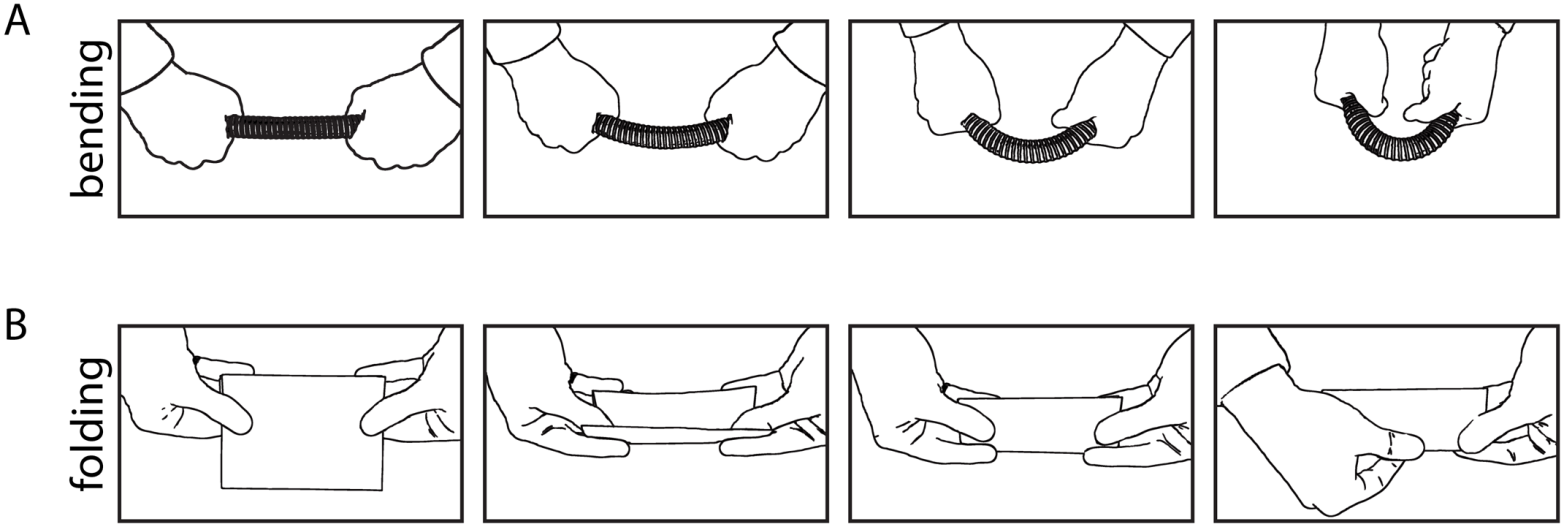
Example of typical sequences of hand and finger movements while applying a (A) bending or (B) folding transformation.

Also, note that physical transformations are defined by a sequence of forces applied to an object. We suggest that for manual execution, this sequence is rather stereotypical so that in addition to identifying shape features that are typical for a particular transformation, it may also be possible to identify the sequence of hand and finger movements together with the applied forces that are typically used to realize a transformation. In other words, in addition to identifying the class of transformation, the observer may also be able to recover the causal history sequence [11]. For example, a typical bending transformation involves holding an object at two distant points along its main axis and then moving those together by applying force (Fig 11A), while a typical type of folding involves the material being bent over the two thumbs and then being creased along the resulting fold with thumb and index finger of one hand (Fig 11B). These stereotypical sequences of manual executions might also affect perceived similarity between objects affected by the same transformation: even though shape features of folded paper and steel might be more similar compared to those of folded cloth, the manual actions involved in folding paper and cloth are more similar compared to those involved in folding steel. Consequently, paper and cloth might be judged as being more similar with respect to their causal history compared to steel–in conflict with their relatively dissimilar shape features.

### 5.4 Interactions of perceiving materials and transformations

The inference of causal history is also affected by the material of the objects. Previous findings suggest that the perceived material identity of objects affects judgments about other internal properties of these objects (e.g., [28–31]). Here, we show that perceived material also affects judgments about external properties, that is, about the causal history of objects. We find an interaction between object material and the ability to identify the physical transformation (but not so much vice versa). For example, in Experiment 2, folded objects are perceived more often as bent when the material is putty or wire compared to other materials. In Experiment 3, this is even more pronounced: bent and folded objects are often confused, and the pattern of these confusions vary with the object material–with objects made from wire been confused most often. It is important to note that we made no attempt to standardize the transformations applied to different samples. The materials were simply shaped by hand, with the understanding that different samples and different materials would respond in different ways, leading to different cues in the stimuli. Indeed, to the extent that there are differences between materials, these are presumably driven primarily by stimulus differences, rather than some fundamental difference in the subjects’ inference skills for different materials.

These findings fit well within our scheme of explaining the identification of transformations via particular shape features: transformation judgments depend on the material specifically when (i) the type of material affects the extent to which the typical shape features associated with a transformation are expressed, and (ii) the type of material makes it difficult to see the shape features.

First, the same physical transformation (i.e., the same external forces applied) can lead to different shape features being expressed: A bending transformation can easily produce shape features typical for folding if the physical properties of the material do not allow it to transform smoothly (i.e., in foil or cardboard, a bending transformation will often produce distinct folds; Fig 10, third row). In the limits, not all transformations can be applied to all objects, for example, a board of timber cannot be crumpled. This implies that the mappings between transformation categories and shape features cannot be universal. As suggested above, folding paper, cloth, and steel will produce different shape features even though all belong to the same transformation category. This is the same problem that we are facing in material perception, where very different color and texture features can map unto the same material category (e.g., different kinds of plastic, wood or glass; [32]). For a strict test of this particular type of interaction between materials and transformations, future studies will have to define transformations in terms of their manual execution and forces. For example, it could be tested to what extent different materials are affected by exactly the *same* transformation (for a computer simulation approach to testing the effect of the same force unto an object with different internal properties see [29]). In the current study, we did not strictly control the transformation execution as we reasoned that would be more natural: humans also vary the procedures and forces when applying manual transformations depending on the object material and material properties to produce the desired effects (e.g., to twist a piece of paper they will use less force compared to chicken wire to avoid tearing the paper).

Second, the material itself can make it harder to see the shape features: in an object made from wire it is much harder to identify local features compared to global features–thus, folded and bent objects are more easily confused (Fig 10, fourth row) and twisted objects are mislabeled as being bent when this is suggested by their global shape (e.g., third object in first row of Fig 10). Note that for the same reason, judgments about transformations are also affected by viewpoint–for example, when the typical shape features can just not be seen from the current viewpoint (e.g., first object in third row of Fig 10).

### 5.5 Conclusion

We asked participants to make inferences about the causal history of real objects from photographs. Objects were made of different materials and were transformed by either twisting, crumpling, bending, or folding them. We show that participants perform far above chance, thereby providing–to our knowledge for the first time–direct evidence for the visual inference of causal history from real objects. Of course, our findings and conclusions are limited by the selection of materials and transformations, and will not generalize to all other materials and transformations. For example, our materials stem from a class of ‘smoothly transforming’ materials–we did not include any material that would break or splinter in response to physical transformation. In the same way, we could have included other transformations, such as ripping or hammering, that would give rise to other specific shape features. Still, we are confident that our findings illustrate our general capabilities for understanding shape by parsing and interpreting causally significant features, and thereby recovering the causal history of objects. This inference is important–as knowing the causal history of a given object will affect other perceptual and cognitive tasks, for example, (i) object constancy–by identifying objects across transformations; (ii) predictions of object behavior–by knowledge about previous transformations; (iii) categorization–by using transformations as common denominator of categories; or (iv) predictions of plausible variants–by applying transformations to other objects through mental imagery.

## Supporting information

S1 Fig Raw free naming data from Experiment 1. The different panels show naming responses for the different (A) materials, and (B) transformations. The bars plot the frequency of naming responses [percent] with the actual material/transformation in black.
(PDF)

S1 Table Paired t-tests comparing ratings between different materials in the material rating task. ** indicates p < .001 and * indicates p < .05.
(PDF)

S2 Table Paired t-tests comparing ratings between different transformations in the material rating task. ** indicates p < .001 and * indicates p < .05.
(PDF)

S3 Table Paired t-tests comparing ratings between different transformations in the transformation rating task. ** indicates p < .001 and * indicates p < .05.
(PDF)

S4 Table Paired t-tests comparing ratings between different materials in the transformation rating task. ** indicates p < .001 and * indicates p < .05.
(PDF)

S5 Table Paired t-tests comparing ratings between different materials in the transformation rating task within the class of bend objects. ** indicates p < .001 and * indicates p < .05.
(PDF)

S6 Table Paired t-tests comparing ratings between different transformations in the 4-AFC task. ** indicates p < .001 and * indicates p < .05.
(PDF)

S7 Table Paired t-tests comparing ratings between different materials in the transformation rating task. ** indicates p < .001 and * indicates p < .05.
(PDF)

S8 Table Paired t-tests comparing ratings between different materials in the transformation rating task within the class of bend objects. ** indicates p < .001 and * indicates p < .05.
(PDF)

S9 Table Paired t-tests comparing ratings between different materials in the transformation rating task within the class of folded objects. ** indicates p < .001 and * indicates p < .05.
(PDF)
