# Supplementary material for: Identifying shape transformations from photographs of real objects
Source: PLoS One. 2018 Aug 16;13(8):e0202115. doi: 10.1371/journal.pone.0202115 (PMC6095529; doi:10.1371/journal.pone.0202115)
Supplement: S1 Fig — The different panels show naming responses for the different (A) materials, and (B) transformations. The bars plot the frequency of naming responses [percent] with the actual material/transformation in black. (PDF) [file pone.0202115.s001.pdf]

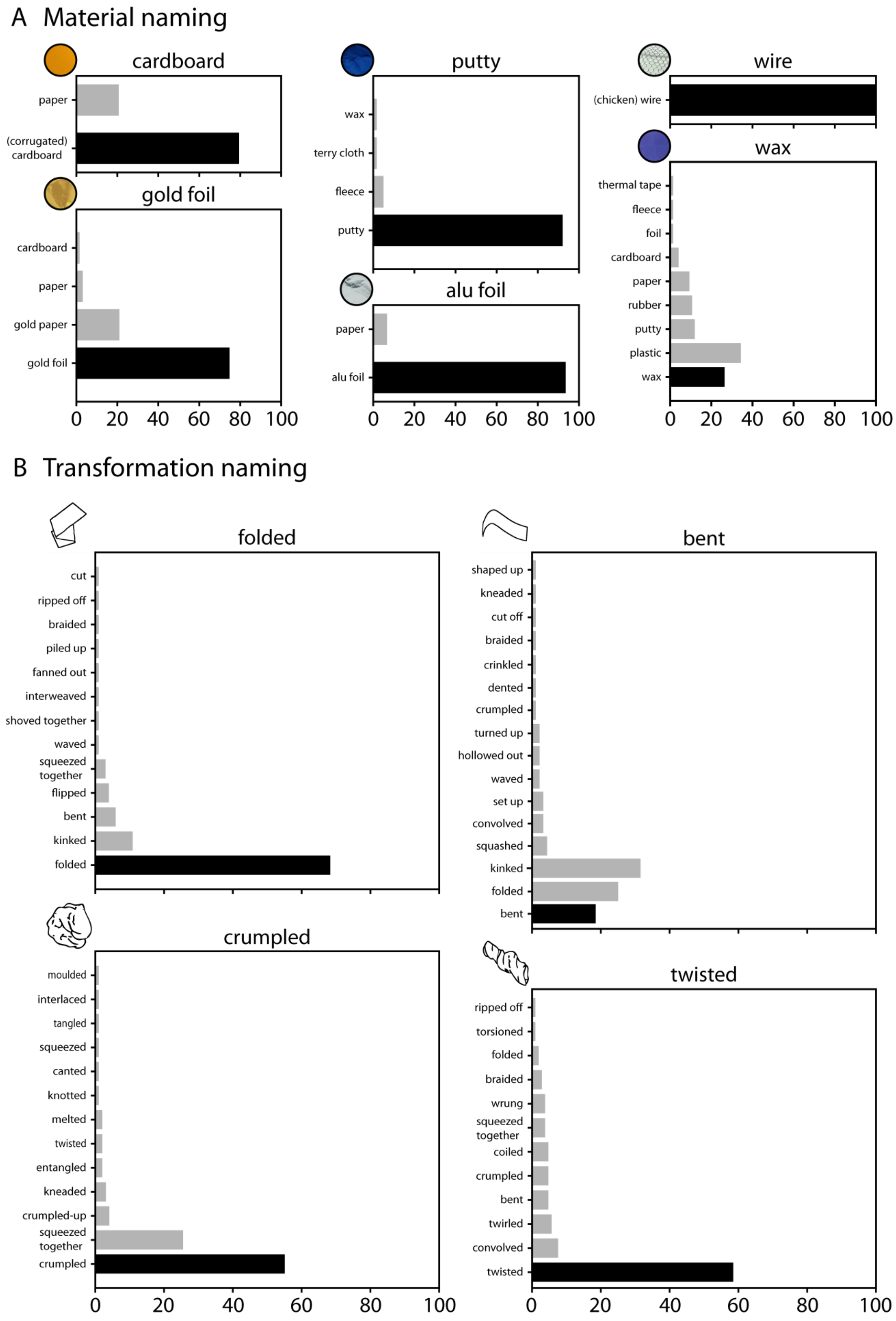

S1 Fig. Raw free naming data from Experiment 1. The different panels show naming responses for the different (A) materials, and (B) transformations. The bars plot the frequency of naming responses [percent] with the actual material/transformation in black.
