# Supplementary material for: Identifying shape transformations from photographs of real objects
Source: PLoS One. 2018 Aug 16;13(8):e0202115. doi: 10.1371/journal.pone.0202115 (PMC6095529; doi:10.1371/journal.pone.0202115)
Supplement: S1 Table — ** indicates p < .001 and * indicates p < .05. (PDF) [file pone.0202115.s002.pdf]

S1 Table. Paired t-tests comparing ratings between different materials in the material rating task.

comparison		*T*	*df*	*p*
cardboard	cardboard	NaN	NaN	NaN
cardboard	putty	0.14	14	.893
cardboard	wire	-0.74	14	.470
cardboard	gold foil	0.26	14	.798
cardboard	aluminium foil	0.60	14	.559
cardboard	wax	20.97	14	.055
putty	putty	NaN	NaN	NaN
putty	wire	-0.54	14	.595
putty	gold foil	0.12	14	.903
putty	aluminium foil	0.57	14	.577
putty	wax	20.87	14	.056
wire	wire	NaN	NaN	NaN
wire	gold foil	0.92	14	.374
wire	aluminium foil	0.85	14	.408
wire	wax	21.37	14	.051
gold foil	gold foil	NaN	NaN	NaN
gold foil	aluminium foil	0.29	14	.779
gold foil	wax	20.44	14	.060
aluminium foil	aluminium foil	NaN	NaN	NaN
aluminium foil	wax	19.27	14	.075
wax	wax	NaN	NaN	NaN

** indicates $p < .001$ and * indicates $p < .05$
