# Supplementary material for: Identifying shape transformations from photographs of real objects
Source: PLoS One. 2018 Aug 16;13(8):e0202115. doi: 10.1371/journal.pone.0202115 (PMC6095529; doi:10.1371/journal.pone.0202115)
Supplement: S2 Table — ** indicates p < .001 and * indicates p < .05. (PDF) [file pone.0202115.s003.pdf]

S2 Table. Paired t-tests comparing ratings between different transformations in the material rating task.

comparison		*T*	*df*	*p*
folded	folded	NaN	NaN	NaN
folded	bent	11.23	14	.280
folded	crumpled	0.94	14	.364
folded	twisted	0.55	14	.592
bent	bent	NaN	NaN	NaN
bent	crumpled	0.25	14	.809
bent	twisted	-12.05	14	.248
crumpled	crumpled	NaN	NaN	NaN
crumpled	twisted	-0.80	14	.437
twisted	twisted	NaN	NaN	NaN

** indicates p < .001 and * indicates p < .05
