# Supplementary material for: Identifying shape transformations from photographs of real objects
Source: PLoS One. 2018 Aug 16;13(8):e0202115. doi: 10.1371/journal.pone.0202115 (PMC6095529; doi:10.1371/journal.pone.0202115)
Supplement: S3 Table — ** indicates p < .001 and * indicates p < .05. (PDF) [file pone.0202115.s004.pdf]

S3 Table. Paired t-tests comparing ratings between different transformations in the transformation rating task.

comparison		*T*	*df*	*p*
folded	folded	NaN	NaN	NaN
folded	bent	52.57	14	.000**
folded	crumpled	-26.22	14	.020*
folded	twisted	-29.61	14	.010*
bent	bent	NaN	NaN	NaN
bent	crumpled	-57.03	14	.000**
bent	twisted	-55.99	14	.000**
crumpled	crumpled	NaN	NaN	NaN
crumpled	twisted	-0.39	14	.702
twisted	twisted	NaN	NaN	NaN

** indicates p < .001 and * indicates p < .05
