# Supplementary material for: Identifying shape transformations from photographs of real objects
Source: PLoS One. 2018 Aug 16;13(8):e0202115. doi: 10.1371/journal.pone.0202115 (PMC6095529; doi:10.1371/journal.pone.0202115)
Supplement: S4 Table — ** indicates p < .001 and * indicates p < .05. (PDF) [file pone.0202115.s005.pdf]

S4 Table. Paired t-tests comparing ratings between different materials in the transformation rating task.

comparison		*T*	*df*	*P*
cardboard	cardboard	NaN	NaN	NaN
cardboard	putty	30.22	14	.009*
cardboard	chicken wire	21.56	14	.049*
cardboard	gold foil	24.57	14	.028*
cardboard	aluminium foil	30.52	14	.009*
cardboard	wax	-11.67	14	.263
putty	putty	NaN	NaN	NaN
putty	chicken wire	-19.02	14	.078
putty	gold foil	-20.97	14	.055
putty	aluminium foil	-14.81	14	.161
putty	wax	-41.39	14	.001*
chicken wire	chicken wire	NaN	NaN	NaN
chicken wire	gold foil	-0.83	14	.420
chicken wire	aluminium foil	-0.40	14	.692
chicken wire	wax	-30.23	14	.009*
gold foil	gold foil	NaN	NaN	NaN
gold foil	aluminium foil	0.98	14	.343
gold foil	wax	-43.03	14	.000**
aluminium foil	aluminium foil	NaN	NaN	NaN
aluminium foil	wax	-40.26	14	.001*
wax	wax	NaN	NaN	NaN

** indicates $p < .001$ and * indicates $p < .05$
