# Supplementary material for: Identifying shape transformations from photographs of real objects
Source: PLoS One. 2018 Aug 16;13(8):e0202115. doi: 10.1371/journal.pone.0202115 (PMC6095529; doi:10.1371/journal.pone.0202115)
Supplement: S5 Table — ** indicates p < .001 and * indicates p < .05. (PDF) [file pone.0202115.s006.pdf]

S5 Table. Paired t-tests comparing ratings between different materials in the transformation rating task within the class of bend objects.

comparison		*T*	*df*	*P*
cardboard	cardboard	NaN	NaN	NaN
cardboard	putty	0.67	14	.511
cardboard	chicken wire	-13.11	14	.211
cardboard	gold foil	22.23	14	.043*
cardboard	aluminium foil	51.25	14	.000**
cardboard	wax	-0.55	14	.590
putty	putty	NaN	NaN	NaN
putty	chicken wire	-27.57	14	.015*
putty	gold foil	11.03	14	.289
putty	aluminium foil	25.11	14	.025*
putty	wax	-15.03	14	.155
chicken wire	chicken wire	NaN	NaN	NaN
chicken wire	gold foil	34.03	14	.004*
chicken wire	aluminium foil	50.99	14	.000**
chicken wire	wax	0.97	14	.350
gold foil	gold foil	NaN	NaN	NaN
gold foil	aluminium foil	36.09	14	.003*
gold foil	wax	-28.72	14	.012*
aluminium foil	aluminium foil	NaN	NaN	NaN
aluminium foil	wax	-49.22	14	.000**
wax	wax	NaN	NaN	NaN

** indicates $p < .001$ and * indicates $p < .05$
