# Supplementary material for: Identifying shape transformations from photographs of real objects
Source: PLoS One. 2018 Aug 16;13(8):e0202115. doi: 10.1371/journal.pone.0202115 (PMC6095529; doi:10.1371/journal.pone.0202115)
Supplement: S6 Table — ** indicates p < .001 and * indicates p < .05. (PDF) [file pone.0202115.s007.pdf]

S6 Table. Paired t-tests comparing ratings between different transformations in the 4-AFC task.

comparison		***T***	***df***	***p***
folded	folded	NaN	NaN	NaN
folded	bent	45.26	14	.000**
folded	crumpled	-47.18	14	.000**
folded	twisted	-43.77	14	.000**
bent	bent	NaN	NaN	NaN
bent	crumpled	-60.93	14	.000**
bent	twisted	-62.66	14	.000**
crumpled	crumpled	NaN	NaN	NaN
crumpled	twisted	13.13	14	.210
twisted	twisted	NaN	NaN	NaN

** indicates p < .001 and * indicates p < .05
