# Supplementary material for: Identifying shape transformations from photographs of real objects
Source: PLoS One. 2018 Aug 16;13(8):e0202115. doi: 10.1371/journal.pone.0202115 (PMC6095529; doi:10.1371/journal.pone.0202115)
Supplement: S7 Table — ** indicates p < .001 and * indicates p < .05. (PDF) [file pone.0202115.s008.pdf]

S7 Table. Paired t-tests comparing ratings between different materials in the transformation rating task.

comparison		*T*	*df*	*p*
cardboard	cardboard	NaN	NaN	NaN
cardboard	putty	16.96	14	.112
cardboard	chicken wire	20.09	14	.064
cardboard	gold foil	18.76	14	.082
cardboard	aluminium foil	47.12	14	.000**
cardboard	wax	0.47	14	.647
putty	putty	NaN	NaN	NaN
putty	chicken wire	0.21	14	.839
putty	gold foil	-0.75	14	.466
putty	aluminium foil	10.68	14	.304
putty	wax	-14.18	14	.178
chicken wire	chicken wire	NaN	NaN	NaN
chicken wire	gold foil	-12.43	14	.234
chicken wire	aluminium foil	11.46	14	.271
chicken wire	wax	-22.41	14	.042*
gold foil	gold foil	NaN	NaN	NaN
gold foil	aluminium foil	30.77	14	.008*
gold foil	wax	-17.36	14	.105
aluminium foil	aluminium foil	NaN	NaN	NaN
aluminium foil	wax	-38.91	14	.002*
wax	wax	NaN	NaN	NaN

** indicates p < .001 and * indicates p < .05
