# Supplementary material for: Identifying shape transformations from photographs of real objects
Source: PLoS One. 2018 Aug 16;13(8):e0202115. doi: 10.1371/journal.pone.0202115 (PMC6095529; doi:10.1371/journal.pone.0202115)
Supplement: S8 Table — ** indicates p < .001 and * indicates p < .05. (PDF) [file pone.0202115.s009.pdf]

S8 Table. Paired t-tests comparing ratings between different materials in the transformation rating task within the class of bend objects.

comparison		*T*	*df*	*p*
cardboard	cardboard	NaN	NaN	NaN
cardboard	putty	-29.47	14	.011*
cardboard	chicken wire	-34.72	14	.004*
cardboard	gold foil	14.47	14	.170
cardboard	aluminium foil	61.73	14	.000**
cardboard	wax	0.65	14	.526
putty	putty	NaN	NaN	NaN
putty	chicken wire	0.96	14	.355
putty	gold foil	38.33	14	.002*
putty	aluminium foil	61.24	14	.000**
putty	wax	35.65	14	.003*
chicken wire	chicken wire	NaN	NaN	NaN
chicken wire	gold foil	45.30	14	.000**
chicken wire	aluminium foil	75.21	14	.000**
chicken wire	wax	46.00	14	.000**
gold foil	gold foil	NaN	NaN	NaN
gold foil	aluminium foil	45.72	14	.000**
gold foil	wax	-11.84	14	.256
aluminium foil	aluminium foil	NaN	NaN	NaN
aluminium foil	wax	-54.41	14	.000**
wax	wax	NaN	NaN	NaN

** indicates $p < .001$ and * indicates $p < .05$
