# Supplementary material for: Identifying shape transformations from photographs of real objects
Source: PLoS One. 2018 Aug 16;13(8):e0202115. doi: 10.1371/journal.pone.0202115 (PMC6095529; doi:10.1371/journal.pone.0202115)
Supplement: S9 Table — ** indicates p < .001 and * indicates p < .05. (PDF) [file pone.0202115.s010.pdf]

S9 Table. Paired t-tests comparing ratings between different materials in the transformation rating task within the class of folded objects.

comparison		*T*	*df*	*p*
cardboard	cardboard	NaN	NaN	NaN
cardboard	putty	43.28	14	.000**
cardboard	chicken wire	37.14	14	.002*
cardboard	gold foil	30.20	14	.009*
cardboard	aluminium foil	-10.31	14	.320
cardboard	wax	-1.00	14	.334
putty	putty	NaN	NaN	NaN
putty	chicken wire	-0.98	14	.345
putty	gold foil	-41.06	14	.001*
putty	aluminium foil	-46.09	14	.000**
putty	wax	-44.38	14	.000**
chicken wire	chicken wire	NaN	NaN	NaN
chicken wire	gold foil	-30.98	14	.008*
chicken wire	aluminium foil	-39.08	14	.002*
chicken wire	wax	-40.26	14	.001*
gold foil	gold foil	NaN	NaN	NaN
gold foil	aluminium foil	-47.99	14	.000**
gold foil	wax	-43.80	14	.000**
aluminium foil	aluminium foil	NaN	NaN	NaN
aluminium foil	wax	0.46	14	.653
wax	wax	NaN	NaN	NaN

** indicates $p < .001$ and * indicates $p < .05$
